# The Surgeon’s Blind Spot: A Narrative Review of Psychological Risks and Assessment Strategies in Invasive Aesthetic Surgery Patients

**DOI:** 10.7759/cureus.99284

**Published:** 2025-12-15

**Authors:** Harmeen Jagpal

**Affiliations:** 1 Psychiatry, Queen Elizabeth Hospital Birmingham, Birmingham, GBR

**Keywords:** aesthetic facial surgery, aesthetic surgery, anxiety, body dysmorphia, body image, cosmetic procedures, facial cosmetic surgery, self-esteem, suicide and depression

## Abstract

Cosmetic surgery aims to enhance appearance and psychological well-being, and its popularity is rapidly increasing. Cosmetic surgery patients are much more likely to report mental health concerns than other surgical groups, yet clinicians often lack understanding of psychological outcomes and assessment strategies. A literature search was performed using PubMed, MEDLINE (Medical Literature Analysis and Retrieval System Online), and Google Scholar with the keywords “cosmetic surgery”, “aesthetic surgery”, “self-esteem”, “anxiety”, and “body dysmorphia” to evaluate the psychological effects of invasive cosmetic procedures. Additionally, this review evaluates the use of pre-operative psychological assessments within the United Kingdom cosmetic surgery practices. Most studies showed cosmetic surgery improved body image but had limited or no effect on self-esteem. Depression often persisted or worsened postoperatively. Women undergoing breast augmentation were two to three times more likely to die by suicide than other cosmetic surgery patients. Individuals with body dysmorphic disorder (BDD) consistently experienced no improvement or a decline in psychological well-being after surgery. Pre- and post-operative psychological assessment is essential but underused, with over half of the plastic surgeons in the United Kingdom not routinely screening patients. Standardised assessment guidelines are urgently needed to mitigate psychological harm.

## Introduction and background

The popularity of aesthetic surgery is rapidly increasing, fueled by social media’s influence on beauty standards. According to the British Association of Aesthetic Plastic Surgeons, 31,057 cosmetic procedures took place in 2022 in the United Kingdom (UK), marking a 102% rise from the previous year. Women accounted for 93% of these procedures, with breast augmentation, breast reduction, abdominoplasty, liposuction, and blepharoplasty being among the most common [1]. 

Aesthetic surgeons encounter patients with psychological vulnerabilities more frequently than other surgical specialities. The history of mental illness was found in 19% of patients undergoing aesthetic surgery, as opposed to 4% of those undergoing non-aesthetic surgery [2]. Thus, cosmetic surgery patients may remain psychologically vulnerable even when postoperative results are satisfactory. Postoperative psychological difficulties can range from increased self-consciousness to major depressive disorder and body dysmorphic disorder (BDD).

Many individuals pursue cosmetic procedures with expectations of psychological or emotional benefit. Given this close relationship between aesthetic surgery and mental well-being, it is essential that the psychological health of every patient is assessed both pre- and postoperatively. Despite this central motivation for undergoing these elective surgeries, medical professionals often have limited knowledge of the psychological effects of aesthetic surgery. This is compounded by the lack of research on effective strategies to minimise the risk of deleterious psychological outcomes. 

This literature review aims to assess the effect of invasive aesthetic surgery on five psychological domains: body image, self-esteem, anxiety and depression, suicide, and BDD. It will be demonstrated that there is evidence to suggest aesthetic surgery has a positive effect on body image, but a mixed effect on complex psychological constructs such as anxiety and self-esteem. There are strong empirical findings to demonstrate that aesthetic surgery may have a deleterious and compound effect on pre-existing mental health challenges. While acknowledging existing reviews on this topic, this review also includes the current application of psychological assessments in aesthetic surgery and explores opportunities for their implementation. 

This article was previously presented as a poster at the Faculties of Child and Adolescent and General Adult Psychiatry Joint Spring Conference on March 18, 2025.

## Review

Materials and methods 

Literature on the psychological impact of cosmetic surgery was reviewed using the electronic databases PubMed, MEDLINE (Medical Literature Analysis and Retrieval System Online), and Google Scholar with the keywords “cosmetic surgery”, “aesthetic surgery”, “self-esteem”, “anxiety”, and “body dysmorphia”. Articles were restricted to those published in the English language. This yielded 4260 articles. After screening the titles/abstracts and excluding duplicates, 30 articles were deemed most appropriate to include in this review. This review is primarily narrative; therefore, no quantitative synthesis was intended/conducted. 

The Royal College of Surgeons England defines cosmetic surgery as an operation performed to alter a person's physical appearance for aesthetic reasons, rather than for medical purposes (in the absence of disease, injury, wound, or congenital deformity) [3]. This review will focus on invasive aesthetic surgery, rather than non-invasive procedures. While there is currently no formal definition of invasive cosmetic surgery, this review defines it as a procedure that alters a body feature by cutting into the skin for aesthetic purposes. Table 1 shows the inclusion and exclusion criteria.

**Table 1:** Inclusion and exclusion criteria used

Inclusion Criteria	Exclusion Criteria
Cosmetic surgeries for aesthetic reasons	Reconstructive surgeries on patients secondary to diseases e.g. breast reconstruction after a therapeutic mammaplasty secondary to breast cancer.
Invasive procedures e.g. rhinoplasty, breast augmentation	Studies on non-invasive cosmetic procedures e.g. Dermal fillers, chemical peels, Botulinum toxin filler
Studies in the English language	Studies not in the English language
Studies published after the year 1995	Studies published before the year 1995
Psychiatric assessment required	Studies that do not include a psychiatric assessment
Prospective cohort studies, case series, cross-sectional surveys, narrative reviews.	Randomised controlled trials, systematic reviews, qualitative studies, case-control studies

Results 

Body Image

Body image is a multidimensional concept involving an individual's perceptions, thoughts, behaviours, and attitudes about their appearance [4]. Most studies utilised the Multi-dimensional Body-Self Relations Questionnaire (MBSRQ) to reveal an improvement in body image. Overall, the evidence suggests that invasive cosmetic procedures tend to enhance body image, though the strength and durability of these effects vary depending on the procedure. 

Sarwer et al., for example, reported long-standing improvements among patients from eight cosmetic surgery practices using repeated MBSRQ at three, six, 12, and 24 months postoperatively [5]. Of the 100 patients in their study, 65 completed the two-year assessment. Improvements in body image observed at three months after surgery were maintained for up to two years, with 78% of patients reporting being ‘extremely satisfied’. However, their study was limited by the fact that 35 patients who did not remain in the study were not investigated. While it is possible that they may have reported similar outcomes as the above, it is also likely that they had a range of experiences, both positive and negative, that were not captured in the study. 

Similar improvements were observed in studies focusing on breast augmentation [6-8]. Murphy et al. demonstrated that improvement in body image persisted over a six-year postoperative period [8], and other prospective cohorts echoed these findings [6,7]. Alderman et al. conducted a study of 14,514 breast implant patients to assess the change in body image [9]. They used a patient-reported outcome measure called the BREAST-Q to assess psychological well-being and body image at postoperative years 1 and 4. They similarly found body image improved in this patient group in parallel with their psychological well-being. This improvement was sustained throughout the four years. Murphy et al. noted that this data may not be broadly generalisable, as the surgical benefits might relate specifically to feelings about the breasts rather than global body image [8]. 

Findings in rhinoplasty patients were more mixed. One study reported increased body image concerns at three months postoperatively [10]. This could be explained by an inverse relationship between body image and postoperative complications [11]. The short follow-up period in Ghazizadeh Hashemi's study [10] may not be reliable to assess post-rhinoplasty body image concerns, as the final nasal tip shape can take up to six months to show. The immediate nasal swelling and bruising could explain the increased body image concerns in their study. Despite the time limit of their study, it was one of the few studies that assessed male patients (24 patients were female, whilst 36 were male) and showed no significant relationship between sex and change in body image. Long-term studies provide clearer evidence: Cingi et al. found improvements in appearance orientation at one year post surgery [12]. Klassen et al., with a long follow-up of three years, demonstrated overall body image improvement after rhinoplasty [13]. Rhinoplasty outcomes suggest that benefits may emerge later than in other procedures, highlighting the importance of adequate follow-up periods.

The majority of studies showed an improvement in body image despite the type of cosmetic procedure conducted. A summary of the studies reviewed in this section is provided in Table 2. 

**Table 2:** Summary of studies examining the impact of cosmetic surgery on body image

Study	Number of Participants	Follow-up Period	Outcome and Screening Tool(s) used	Type of Cosmetic Surgery Performed
Sarwer et al. (2008) [5]	100	2 years	Improvement in body image at 3 months after surgery was maintained up to 2 years. The screening tool(s) used were not specified.	Variety of procedures, including breast augmentation and blepharoplasty
Banbury et al. (2004) [6]	47	6 months	Improvement in body image at 3 and 6 months post-surgery. The Multidimensional Body-Self Relations Questionnaire was used.	Breast Augmentation
Sarwer et al. (2005) [7]	100	1 year	Improvement in body image at 3 months after surgery was maintained up to 1 year. The screening tool(s) used were not specified.	Variety of procedures, including breast augmentation and blepharoplasty
Murphy et al. (2009) [8]	455	6 years	Improvement in sexual attractiveness and body image was maintained up to 6 years. The screening tool(s) used were not specified.	Breast Augmentation
Ghazizadeh Hashemi et al. (2017) [10]	60	3 months	Increase in concerns regarding body image. The Body Image Concern Inventory (BICI) was used.	Rhinoplasty
Cingi et al. (2011) [12]	225	1 years	Post-surgical improvements in body image. European QOL Questionnaire (EQ), Rhinoplasty Outcomes Evaluation Questionnaire (ROE), and the Multidimensional Body-Self Relations Questionnaire (MBSRQ) were used.	Rhinoplasty
Alderman et al .(2016) [9]	14,514	4 years	Improvement in body image at 1 year and 4 years after surgery. BREAST-Q was used.	Breast Augmentation
Klassen et al. (2016) [13]	23	3 years	Improvement in body image of the nose, nostrils, and face. FACE-Q was used.	Rhinoplasty

Self-Esteem

Self-esteem, defined as a person's subjective sense of overall personal worth or value, showed a more complex pattern in the literature. While cosmetic surgery often enhances satisfaction with specific body areas, its effect on global self-esteem appears less consistent. 

Swanson’s study exemplifies this nuance: 89% of breast reduction patients reported an improvement in self-esteem, an effect likely tied not only to aesthetic enhancement but also to relief from physical symptoms such as back, shoulder, and neck pain [14]. The primary reason for breast reduction surgery reported in their study was a combination of aesthetic concerns and physical discomfort. Patients reported an improved quality of life due to aesthetic satisfaction as well as increased physical well-being, improved breathing, and reduced pain, which subsequently enhanced their self-worth and self-esteem. However, patients undergoing other types of cosmetic surgeries did not report a moderate or large improvement in self-esteem. Von Soest et al. found that 89% of patients were more than satisfied with their general appearance five years postoperatively, but only small improvements in self-esteem were noted [15]. The results align with other studies indicating that while cosmetic surgery improves self-satisfaction with appearance, larger psychological constructs such as self-esteem remain largely unaffected [16]. Thus, self-esteem is a multi-factorial construct that is not solely based on self-satisfaction with appearance. Many other factors, such as personal achievements, relationships, competence, and self-acceptance, contribute to an individual’s sense of self-esteem. This is supported by Akhlaghi et al., who found that augmentations in an individual’s self-esteem postoperatively were often dependent on the patient’s psychological traits [17]. They found that subjects with identity moratorium reported a significant reduction in self-esteem post cosmetic surgery, whereas subjects who had a susceptible identity psychologically reported an improvement in self-esteem.

Women have reported higher sexual self-esteem after cosmetic surgery, but empirical data suggest that this effect is dependent on two key factors: the type of procedure performed and the cultural context [18]. For instance, rhinoplasty did not significantly impact sexual self-esteem in women from Western countries [19]. Studies in these regions found that the primary determinant of improved sexual self-esteem was cosmetic surgery targeting sexual organs. In contrast, research from more conservative regions, such as Iran, indicated that procedures like rhinoplasty could lead to an increase in sexual self-esteem [20]. This suggests that cultural norms and values, particularly in more conservative societies, may play a significant role in how cosmetic procedures affect one's sense of self-esteem and body image.

Therefore, while cosmetic surgery can enhance certain aspects of self-worth, it is not a comprehensive solution for improving one’s overall self-esteem. A summary of the studies reviewed in this section is provided in Table 3.

**Table 3:** Summary of studies examining the impact of cosmetic surgery on self-esteem

Study	Number of Participants	Follow-up Period	Outcome and Screening Tool(s) used	Type of Cosmetic Surgery Performed
Swanson (2013) [14]	106	1 month	Improvement in self-esteem. Custom questionnaires were used.	Vertical mastopexy, augmentation/mastopexy, and breast reduction.
Von Soest et al. (2011) [15]	130	5 years	A small increase in self-esteem. Custom questionnaires were used.	Variety of procedures, including breast augmentation and blepharoplasty
Esmalian Khamseh et al. (2020) [18]	80	5 months	Improvement in sexual self-esteem. The Sexual Self-Esteem Questionnaire (SSEI-W-SF) was used.	Variety of procedures, including breast augmentation and blepharoplasty
Baniasadi (2012) [19]	30	5 months	No significant improvement in sexual self-esteem. The Multidimensional Body-Self Relations Questionnaire was used.	Rhinoplasty
Najjaran Toussi et al. (2019) [20]	40	1 year	Improvement in sexual self-esteem. Body Image Concern Inventory, Sexual Self-Esteem Index for Women and Body Exposure during Sexual Activities Questionnaire	Rhinoplasty
Bashizadeh et al. (2017) [16]	60	Unknown	No significant improvement in self-esteem. Rosenberg self-esteem scale was used alongside custom questionnaires.	Blepharoplasty
Akhlaghi et al. (2015) [17]	46	4 months	Self-esteem improved in patients with a susceptible identity. An Ego Identity Status - Self Esteem Questionnaire was used.	Variety of procedures, including breast augmentation and blepharoplasty

Depressive Symptoms

Two studies showed no change or a worsening in depressive symptoms after cosmetic surgery [21,22]. Meningaud et al. assessed 79 patients pre- and post-facial cosmetic surgeries [21]. They assessed depressive symptoms with the Montgomery and Asberg Depression Rating Scale (MADRS) and found no significant changes in depressive symptoms nine months post surgery; however, they did observe an improvement in other mental health conditions, such as anxiety. 

Cosmetic surgery, often sought to enhance self-esteem and body image, can paradoxically contribute to increased depression and suicide risk. A strong link between breast augmentation and suicide has been identified in numerous studies. It’s been found that women undergoing breast augmentation are two to three times more likely to commit suicide than those undergoing other cosmetic surgery procedures [23]. Jacobsen et al. studied 2761 Danish women who underwent breast augmentation and compared them with 1736 women who underwent other types of cosmetic surgeries [24]. They discovered that women who underwent breast augmentation had a higher rate of death by suicide, and 8% of women who underwent breast augmentation had a history of psychiatric admission, compared to 5% of women who underwent other forms of cosmetic surgery. 

Pre-disposing factors of this patient group need to be considered when assessing the relationship between suicide and breast augmentation surgery. Women undergoing breast augmentation tend to have higher levels of alcohol and tobacco use and face an increased risk of psychiatric hospital admissions. These are independent risk factors of suicide and suggest that the individuals undergoing breast augmentation surgery are predisposed to mental health conditions. The mental stability of patients undertaking these procedures is further compounded by the psychological distress resulting from postoperative complications and dissatisfaction. Thus, cosmetic procedures do not always improve psychological well-being and may, as suggested by the studies provided in Table 4, exacerbate pre-existing mental health struggles.

**Table 4:** Summary of studies examining the impact of cosmetic surgery on depression, anxiety and suicide

Study	Number of Participants	Follow-up Period	Outcome and Screening Tool(s) used	Type of Cosmetic Surgery Performed
Meningaud et al. (2003) [21]	79	9 months	No change in depressive symptoms, anxiety improved. The Montgomery and Asberg depression rating scale (MADRS), the self-assessment test of thoughts in social interaction (SISST) and the European quality of life 5 dimensions (EQ-5D) were used.	Facial cosmetic surgery
von Soest et al. (2012) [22]	71	13 months	Significant deterioration of anxiety and depression postoperatively. The screening tool(s) used were not specified.	Variety of procedures, including breast augmentation and blepharoplasty
Jacobsen et al. (2004) [24]	2761	/	Increased risk of suicide amongst breast implant patients. A screening tool was not used.	Breast augmentation

Body Dysmorphic Disorder

BDD is a preoccupation with one or more perceived defects or flaws in physical appearance that are not observable to others, causing clinically significant distress or impairment in social, occupational, or other areas of functioning [25]. Many patients with BDD undergo cosmetic surgery to ‘fix’ a perceived physical flaw. All the studies reviewed showed cosmetic surgery had no or even a negative effect on people with BDD. 

A study of 50 cases of BDD patients showed 81% were dissatisfied with the result of cosmetic surgery [26]. These findings could be explained by unrealistic expectations and the nature of BDD. Patients with BDD have a distorted perception of themselves, which is rooted in obsessive thoughts and beliefs. Many people with BDD have cognitive biases that lead them to interpret visual information more negatively. Patients with BDD tend to focus on imperfections that aren’t necessarily a ‘real’ flaw but rather a symptom of the disorder. Thus, even if they undergo cosmetic surgery to 'fix' one perceived flaw, the obsession tends to shift elsewhere, as the underlying issue is their perception and not their actual appearance.

Similarly, Tignol et al. found 85% of BDD patients who had undergone cosmetic surgery still experienced BDD postoperatively, but three patients who did not have BDD preoperatively had interestingly developed BDD at follow-up [27]. Di Mattei et al. [28] and Geliebter et al. [29] further supported the finding that patients with BDD in the preoperative stage continued to have BDD postoperatively. 

Cosmetic surgery can result in an unhealthy cycle as it can reinforce the idea that physical appearance is paramount, causing patients to become hyper-focused on appearance, increasing scrutiny of other body parts, and creating a cycle of dissatisfaction. As aforementioned, when unrealistic expectations aren’t met, patients can be dissatisfied as they feel that corrective surgery hasn’t led to the desired result, fueling the disorder. BDD patients rarely benefit from cosmetic surgery as it does not substantially modify their alterations of body perception. This provides further impetus for mental health screening preoperatively. A summary of the studies reviewed in this section is provided in Table 5.

**Table 5:** Summary of studies examining the impact of cosmetic surgery on body dysmorphic disorder BDDE-SR: Body Dysmorphic Disorder Examination - Self Report

Study	Number of Participants	Follow-up Period	Outcome and Screening Tool(s) used	Type of Cosmetic Surgery Performed
Veale et al. (1996) [26]	50	/	Most patients were dissatisfied with the result. A cross-sectional interview survey was used.	Not specified
Tignol et al. (2007) [27]	24	5 years	Symptoms of BDD were maintained and, for some patients, started after cosmetic surgery. Telephone interviews were used.	Not specified
Di Mattei et al. (2015) [28]	85	12 months	Patients with BDD pre-operatively continued to have symptoms post-operatively. The Body Uneasiness Test, Psychological General Well-Being Index - Short version, and the Glasgow Benefit Inventory were used.	Variety of procedures, including rhinoplasty and breast augmentation
Geliebter et al. (2015) [29]	9	3 months	BDDE-SR scores did not significantly change post-surgery.	Liposuction

Assessments, Limitations, and Future Directions

This review highlights the importance of preoperative and postoperative psychological assessments for cosmetic surgery patients. The preoperative assessment will enable practitioners to identify patients who are susceptible to mental health issues, enabling them to be referred for the appropriate prophylactic treatment/therapy. 

In 2019, the Care Quality Commission (CQC) highlighted the importance of cosmetic surgery patients undergoing psychological assessments, asserting that questionnaires alone were insufficient. However, the CQC did not provide further guidance on how clinicians should carry out these assessments. Dhaliwal et al. found that over 50% of 71 plastic surgeons, working across both the NHS and the private sector, did not use any preoperative psychological tools [30]. This may be due to the limited availability of validated screening tools as well as the challenges posed by their administration, including time and complexity. 

More plastic surgery units could benefit from adopting the psychological assessment framework developed by Clarke et al., which was successfully implemented and evaluated [31]. The model outlined several core requirements, including appearance-related distress, manifestation in behaviour, realistic expectations, and active participation. Standardised measures like the Derriford Appearance Scale (DAS59) were used to assess patients’ psychological state. Their work proved to the department the positive effect of this model, which led to the creation of a permanent psychology position within the department. 

However, more research needs to be conducted on finding the most efficient means to assess the psychological health of cosmetic surgery patients. Ultimately, cosmetic surgery practices need a clear and structured pathway to identify and refer patients suffering from mental health challenges.

A few limitations were identified in this review. Only studies available on electronic databases were used to ease the process of data collection. Therefore, it is difficult to state whether study findings in physical paper journals could have changed the direction of this review. Moreover, no formal risk-of-bias assessment was conducted, limiting the ability to evaluate the methodological quality or internal validity of the studies reviewed.

There was also wide variability in sample sizes, ranging from small cohorts of fewer than 30 participants to large datasets involving thousands of patients. This inconsistency reduces comparability across studies and affects the strength of the conclusions drawn. Cultural and geographic differences among study populations add another layer of variability, as psychological outcomes appeared to differ between Western and non-Western settings. This limits the global generalisability of the findings.

Finally, although cosmetic surgery is increasing in popularity, the vast majority of patients in the included studies were identified as female. The only study that had a reasonable number of male patients was carried out by Cash et al. [11]. Their study found there was no significant difference between sexes on psychological effects. However, more research needs to be conducted on male patients to validate this conclusion. 

As one can see in Tables 2-5, most studies used screening tool questionnaires to assess the effect on the different psychological domains rather than a comprehensive interview approach. Whilst screening tools provide an insight into the effect on body image and self-esteem, these cannot solely be used to assess the prevalence of mental health disorders such as depression and BDD. The author of this review understands that screening tools are easier to conduct logistically, whilst also acknowledging that they cannot be used to arrive at a diagnosis of a psychological disorder. Researchers are still working towards devising the best means to measure psychological changes after appearance modification. Therefore, concrete conclusions cannot be drawn regarding the prevalence of depression and BDD in the post-operative cohort. 

As briefly highlighted above, the studies have also included patients undergoing physical health-related breast reductions, which may closely resemble functional surgery more than cosmetic surgery. Thus, the psychological benefits of breast reduction surgery may be exaggerated due to the physical benefits that patients gain.

## Conclusions

While cosmetic surgery can improve one’s body image by addressing physical features that individuals may find distressing, its positive impact on more complex concepts, such as self-esteem, as well as mental health conditions such as depression and anxiety, is limited. This highlights a critical need for a comprehensive approach to patient care in the field of aesthetic surgery. Currently, the methodologies employed by plastic surgeons to screen patients for psychological suitability for cosmetic procedures vary widely.

More research needs to be conducted to develop evidence-based protocols that identify at-risk individuals before and after surgery. Furthermore, precise and standardised guidelines need to be implemented so that all cosmetic surgery patients receive consistent and holistic care. These guidelines should include psychological assessments and pre- and postoperative counselling. By embedding psychological assessment and evidence-based guidelines into routine practice, the field of aesthetic surgery can improve patient safety, identify vulnerable individuals, and ensure that cosmetic interventions support, not undermine, mental wellbeing.
